# Cold exposure prevents fat accumulation in striped hamsters refed a high-fat diet following food restriction

**DOI:** 10.1186/s40850-022-00122-z

**Published:** 2022-04-18

**Authors:** Da-Liang Huo, Meng-Huan Bao, Jing Cao, Zhi-Jun Zhao

**Affiliations:** 1 College of Life and Environmental Science, Wenzhou University, Wenzhou, 325035 China; 2 State Key Laboratory of Integrated Management for Pest Insects and Rodents, Institute of Zoology, Chinese Academy of Sciences, Beijing, 100101 China

**Keywords:** Cold exposure, Fat accumulation, Metabolic thermogenesis, Striped hamster, Thyroid hormones

## Abstract

**Background:**

In mammals, body mass lost during food restriction is often rapidly regained, and fat is accumulated when *ad libitum* feeding is resumed. Studies in small cold-acclimated mammals have demonstrated significant mobilization of fat deposits during cold exposure to meet the energy requirements of metabolic thermogenesis. However, no studies to our knowledge have examined the effect of cold exposure on fat accumulation during body mass recovery when refed *ad libitum*. In this study, striped hamsters restricted to 80% of their regular food intake were then refed *ad libitum* and exposed to one of three conditions: Intermittent cold temperature (5 °C) for 2 h per day (ICE-2 h/d), intermittent cold temperature (5 °C) for 12 h per day (ICE-12 h/d), or persistent cold exposure (PCE) for four weeks. We measured energy intake, fat deposit mass, serum thyroid hormone levels, and uncoupling protein 1 expression in brown adipose tissue.

**Results:**

There was no significant effect of intermittent or persistent cold exposure on body mass regain, whereas energy intake increased significantly and total fat deposit decreased in the ICE-12 h/d and PCE groups compared to the ICE-2 h/d group and control group maintained at 23 °C (CON). In the ICE-12 h/d and PCE groups, hamsters had 39.6 and 38.3% higher serum 3,3′,5-triiodothyronine levels, respectively, and 81.6 and 71.3% up-regulated expression of uncoupling protein 1, respectively, in brown adipose tissue compared to their counterparts in the CON group. The rate of mitochondrial state III and state IV respiration O_2_ consumption and the activity of cytochrome c oxidase in BAT and liver were significantly higher in the ICE-12 h/d and PCE groups than in the ICE-2 h/d and CON groups.

**Conclusions:**

Our findings suggest thyroid hormone-mediated heat production in brown adipose tissue and liver may be involved in preventing fat accumulation during refeeding in animals frequently or persistently exposed to cold conditions.

**Supplementary Information:**

The online version contains supplementary material available at 10.1186/s40850-022-00122-z.

## Background

Food restriction has been demonstrated to increase lifespan in numerous animals [1–4] and decrease body mass and/or fat deposition in humans and laboratory rodents [5–7]. Body fat loss accounts for most of weight lost during food restriction [8–10]. Previous studies found food-restricted animals had approximately 30% less fat mass and visceral fat compared to those fed *ad libitum*, while their lean body mass remained constant [6, 11]. However, subsequent studies found that the weight was rapidly regained when food was reintroduced *ad libitum* [5, 12, 13], most likely due to compensatory body fat accumulation resulting from a positive energy balance [14–17]. Studies have observed increased levels of hyperphagia in many animals following *ad libitum* refeeding. In contrast, their energy expenditure usually remained unchanged, indicating a positive energy balance due to increased energy intake rather than any changes in energy expenditure.

Increased energy intake in animals with hyperphagia, particularly those eating a high-fat diet, results in obesity due to increased fat accumulation [18–23]. However, in cold conditions, some hyperphagic animals do not accumulate fat [24, 25] because cold-exposed animals must increase thermogenesis to compensate for heat loss, and this requires considerable energy expenditure [25–29]. This suggests that thermogenesis plays a crucial role in maintaining a balance between energy expenditure and fat accumulation. Studies in rodents have demonstrated an enhancing effect of cold exposure on thermogenesis, but the effect of cold exposure on fat accumulation remains inconclusive [27]. For example, there was a significant decrease in white adipose tissue in C57BL/6 mice following sustained cold exposure [30], but not in rats (*Rattus norvegicus*) [31]. Furthermore, researchers have observed varying effects in laboratory mice (*Mus musculus*) subjected to intermittent cold, with no change in fat deposits found for one study [32], and others finding an increase [33, 34] or a decrease [35]. In the wild, small mammals may be exposed more frequently to repetitive or intermittent cold temperatures than to persistent cold temperatures. Thus, it is necessary to examine the effects of repetitive or intermittent cold exposure on body fat deposits.

Changes in thermogenesis at the whole-body level directly reflect changes at the organ/tissue level [36]. Active brown adipose tissue (BAT) burns lipids to produce heat resulting in increased energy expenditure [37]. The production of heat by BAT occurs in the mitochondria due to incomplete coupling between substrate oxidation and ATP production [36–39]. The uncoupling protein 1 (UCP_1_) is predominantly expressed in BAT where it represents approximately 10% of the mitochondrial protein content and mediates a regulated uncoupling process [40]. Studies in small mammals have established that the UCP_1_-based heat production in BAT plays an important role in regulating body temperature [37, 40, 41]. Additionally, BAT heat production effectively reduces adiposity in mice and rats by burning excess energy and playing an important role in promoting fat mobilization and preventing obesity [40, 42–45]. Thyroid hormones (3,3′,5,5′ tetraiodothyronine or T_4_ and 3,3′,5-triiodothyronine or T_3_) are key regulators of thermogenesis, which represents a major component of energy expenditure in homeothermic (‘warm-blooded’) animals [37, 46, 47]. Thyroid hormones are essential for initiating BAT UCP_1_-based heat production as T_3_ potentiates UCP_1_ gene transcription [48–51]. Various animal studies have shown that cold exposure induces a significant increase in metabolic thermogenesis and is linked to increased serum thyroid hormone levels and UCP_1_-based heat production in BAT [27, 42, 52–54]. However, the effects of thyroid and BAT status on energy budgets and fat accumulation during periods of body weight regain following food restriction is currently unknown.

The aim of this study was to investigate the role of thyroid hormone and BAT UCP_1_ in energy intake and fat accumulation in food-restricted striped hamsters refed *ad libitum* and subjected to intermittent versus persistent cold exposure. The striped hamster (*Cricetulus barabensis*) is a common rodent in northern China and is also distributed throughout Russia, Mongolia, and Korea [29]. Striped hamsters do not store food but instead feed on the seeds of crops in winter, and the stems and leaves of plants in summer [55]. Striped hamsters have considerably higher energy intake and metabolic thermogenesis requirements in winter than in summer, yet they have significantly lower fat deposits in winter [56, 57]. Previous studies observed that striped hamsters showed physiological and behavioral strategies to cope with periods of food shortage and regained body weight and fat stores following *ad libitum* feeding [57–60]. In this study, we measured energy intake, fat accumulation, serum T_3_ and T_4_ levels, and BAT UCP_1_ expression in striped hamsters in a cold temperature setting and fed *ad libitum* following a period of food restriction. We hypothesized that when frequently exposed to cold temperatures, striped hamsters would have increased serum thyroid hormone levels and BAT UCP_1_ expression, which would prevent body weight regain and fat accumulation during refeeding.

## Results

### Body mass

There was no significant difference in body mass across the four groups prior to the food restriction treatment (day 1, *F*_3,52_ = 0.28, *P* > 0.05, Fig. 2A). During the food restriction period at 23 °C, body mass decreased by 17.5, 17.7, 18.2 and 18.3% in the CON, ICE-2 h/d, ICE-12 h/d and PCE groups, respectively, on day 21 compared to that on day 7 (day 7–21, *F*_14,42_ = 203.37, *P <* 0.01). However, there was no significant difference in body mass between groups (day 21, *F*_3,52_ = 0.18, *P* > 0.05). Once *ad libitum* feeding was resumed, body mass increased significantly by 15.0, 17.6, 16.1 and 12.1% in the CON, ICE-2 h/d, ICE-12 h/d and PCE groups, respectively, on day 49 compared to that on day 15 (day 15–49, *F*_27,1593_ = 29.97, *P <* 0.01). Body mass did not differ significantly across the four groups at the end of the experiment (day 49, *F*_3,52_ = 1.03, *P* > 0.05, Fig. 2A).

### Food intake

There was no significant difference in food intake across the four groups either before the food restriction treatment (day 1, *F*_3,52_ = 1.952, *P* > 0.05) or during the period of food restriction (day 21, *F*_3,52_ = 1.97, *P* > 0.05, Fig. 2B). Food intake was significantly affected by temperature during the refeeding period, with a significantly higher food intake in the ICE-12 h/d and PCE groups compared to the CON and ICE-2 h/d groups (day 49, *F*_3,52_ = 20.62, *P* < 0.01, Fig. 2B).

### Gross energy intake (GEI) and apparent digestibility

There was a significant difference in GEI across the four groups, which was 50.4 and 92.3% higher in the ICE-12 h/d and PCE groups, respectively, compared to the CON group (*F*_3,51_ = 52.56, *P* < 0.01, post hoc, *P* < 0.01, Fig. 3A). Similarly, DEI was significantly higher in the ICE-12 h/d and PCE groups compared to the CON and ICE-2 h/d groups (*F*_3,51_ = 47.01, *P* < 0.01, post hoc, *P* < 0.01, Fig. 3B). Hamsters in the PCE groups produced 69.8% more feces than those in the CON group (*F*_3,51_ = 36.51, *P* < 0.01, post hoc, *P* < 0.01, Fig. 3C). Apparent digestibility differed significantly across the four groups (*F*_3,51_ = 3.64, *P* < 0.05), being higher in the ICE-12 h/d and PCE groups compared to the CON and ICE-2 h/d groups. However, there was no significant difference in apparent digestibility between each of the three cold exposure groups and the CON group (post hoc, *P* > 0.05, Fig. 3D).

### Fat deposition

BAT mass did not statistically differ across the four groups (*F*_3,52_ = 2.03, *P* > 0.05, Fig. 4A). There were significant differences across the four groups in subcutaneous fat (*F*_3,52_ = 3.32, *P* < 0.05, Fig. 4B), mesentery fat (*F*_3,52_ = 3.99, *P* < 0.05, Fig. 4C), abdominal fat (*F*_3,52_ = 4.72, *P* < 0.01, Fig. 4D) and peritesticular fat (*F*_3,52_ = 3.92, *P* < 0.05), all of which were lower in the PCE group relative to that in the CON or ICE-2 h/d groups (Fig. 4E). Furthermore, total fat mass was significantly different across the four groups (*F*_3,52_ = 3.56, *P* < 0.05, Fig. 4F), with hamsters in the PCE group having less body fat than those in the CON or ICE-2 h/d groups.

### BAT histology

Brown adipocytes in interscapular BAT of hamsters in the ICE-12 h/d and PCE groups had considerably smaller fat droplets than those in the CON group (Fig. 5). Additionally, brown adipocytes in the ICE-12 h/d and PCE groups were noticeably browner than those in the CON and ICE-2 h/d groups.

### Serum thyroid hormones levels and BAT UCP_1_ expression

T_3_ levels were significantly different among the four groups, with the ICE-12 h/d and PCE groups having 39.6 and 38.3% higher T_3_ levels, respectively, than the 23 °C group (*F*_3, 39_ = 3.93, *P* < 0.01, post hoc, *P* < 0.05 or *P* < 0.01, Fig. 6A). However, there was no significant difference in serum T_4_ levels and T_3_/T_4_ ratio between the CON, ICE-2 h/d, ICE-12 h/d and PCE groups (T_4_, *F*_3,39_ = 0.66, *P* > 0.05, Fig. 6B; T_3_/T_4_, *F*_3,39_ = 2.46, *P* > 0.05, Fig. 6C). The expression of BAT UCP_1_ differed significantly across groups, with the ICE-2 h/d, ICE-12 h/d and PCE groups increasing by 34.1, 81.6, and 71.3%, respectively, compared to the CON group (*F*_3,23_ = 14.05, *P* < 0.01, post hoc, *P* < 0.05 or *P* < 0.01, Fig. 6D).

### The rate of state III, state IV and COX activity

The rate of O_2_ consumption of mitochondrial state III and state IV respiration in BAT was significantly different across the four groups, with a significantly higher consumption rate in the PCE group compared to the CON or ICE-2 h/d groups (state III, *F*_3,21_ = 4.22, *P* < 0.05, Fig. 7A; state IV, *F*_3,21_ = 8.86, *P <* 0.01, Fig. 7B). Additionally, BAT COX activity was 116.1 and 102.1% higher in the ICE-12 h/d and PCE groups, respectively, compared to the CON group (*F*_3,21_ = 8.83, *P* < 0.01, post hoc, *P* < 0.01, Fig. 7C). There were also significant increases in the rate of O_2_ consumption of mitochondrial state III and state IV respiration in the liver for the ICE-12 h/d and PCE groups compared to the CON and ICE-2 h/d groups (state III, *F*_3,21_ = 9.79, *P* < 0.05, Fig. 7D; state IV, *F*_3,21_ = 3.40, *P <* 0.05, Fig. 7E). COX activity in the liver was also significantly higher in the PCE group compared to the other three groups (*F*_3,21_ = 8.08, *P <* 0.01, post hoc, *P* < 0.01, Fig. 7F).

## Discussion

In this study, we found that striped hamsters lost weight during food restriction but regained the lost weight and reaccumulated fat stores after being refed *ad libitum*. Interestingly, cold (5 °C) exposure for 12 and 24 h per day did not affect body mass regain, but significantly reduced fat accumulation. Serum T_3_ levels, BAT UCP_1_ expression, and the rate of O_2_ consumption in BAT and liver all increased significantly in the ICE-12 h/d and PCE groups, suggesting that increases in BAT and liver heat production were involved in the prevention of fat accumulation.

Changes in body mass and/or fat mass are important adaptive strategies for small mammals to cope with environmental temperature variations [25, 26, 28, 57, 61–63]. Striped hamsters in this study had a lower body mass and fat content during food restriction but they regained a significant amount of weight when refed *ad libitum*, a pattern similar to that previously reported in other animals [5, 12, 13, 57, 64–66]. Body mass regain did not differ between the ICE-5 h/d, ICE-12 h/d, and PCE groups, and CON group, similar to previous studies that found no significant change in body mass between seasons in wild-caught hamsters [56, 57]. This suggests that body mass regulation of striped hamsters may be independent of cold temperatures, which is notably distinct from other species that show seasonal increases or decreases in body mass [25, 26, 28]. The hamsters in the PCE group consumed significantly more food and therefore might have more chyme in their gastrointestinal tracts than the other three groups. Additionally, the hamsters in the PCE group may gain more lean mass during refeeding to compensate for lower fat deposits. These two possibilities could explain why the different groups of hamsters had the same body mass recovery but differed in fat mass.

Body fat is an essential component in adult organisms that exhibits considerable plasticity in response to environmental variations [6, 11, 16]. Most body mass fluctuations in adult animals, including humans, are usually accounted for by loss or gain of body fat [9, 10, 12, 23]. Inconsistent with body mass, we found that fat accumulation during the *ad libitum* refeeding period was significantly affected by cold exposure. Total fat deposit, which was the sum of subcutaneous, abdominal, mesentery, and peritesticular fat deposits, was 14.1 and 18.2% less in ICE-12 h/d and PCE groups, respectively, than in the CON group. This suggests that changes in body fat are not necessarily consistent with changes in body mass. However, we found that the total fat deposit in the ICE-2 h/d group was not statistically different to that of the CON group. Dulloo et al. (1995) investigated fat deposition in Sprague Dawley male rats (*Rattus norvegicus*) that were refed at room temperature (22 °C), thermoneutrality (29 °C), or cold temperature (6 °C) and found no significant difference in body fat between the three groups [31]. Presby et al. (2019) used intermittent cold exposure (4 °C, 90 min/day, 5 days/wk) to manipulate energy expenditure during maintained weight loss of calorically restricted male FVB mice (*Mus musculus*) [32]. Despite significant increases in energy expenditure during cold exposure, intermittent cold exposure did not affect total daily energy expenditure or fat deposits, likely attributable to compensatory behavior [32]. These findings suggest that among species, cold exposure has a variable effect on fat deposits and that the duration or frequency of cold exposure may influence this effect. In contrast to brief cold exposure, a more frequent or longer duration of cold exposure had a significant effect on preventing fat accumulation in animals that were refed *ad libitum*.

Previous studies have shown that increases in energy intake, or hyperphagia, often leads to fat accumulation, such as high-fat diet-induced obesity [19, 21, 22]. In contrast, a limited or restricted food intake, usually results in fat mobilization in many mammals, including humans [6, 10–12]. Similarly, we found that restricted feeding resulted in significant body weight loss in striped hamsters, which was subsequently regained following *ad libitum* refeeding. Interestingly, striped hamsters in the ICE-12 h/d and PCE groups consumed considerably more food than the control individuals refed *ad libitum* at 23 °C while accumulating significantly less fat. This indicates that frequent or longer duration of cold exposure may prevent fat accumulation in animals that regain their lost weight when food is plentiful. Many small mammals have increased energy intake in response to cold conditions but have lower fat deposits than those in warm conditions [28, 61, 67–69]. These findings demonstrate that energy expenditure may play a more important role in determining fat accumulation than energy intake [55, 70]. However, our findings suggest that the role of energy expenditure in determining fat accumulation may depend on how often the animals are exposed to cold conditions, which can effectively increase energy expenditure, thereby influencing fat accumulation.

Previous studies have shown that BAT and liver are strongly linked to facultative thermogenesis and obligatory thermogenesis, both of which were significantly higher in animals that were exposed or acclimated to cold temperatures [30, 34, 71]. In this study, we found that the rate of oxygen consumption of state III and IV in BAT and liver was significantly higher in the hamsters in the ICE-12 h/d and PCE groups than in the CON group. COX activity was also significantly higher in both tissues in the ICE-12 h/d and PCE groups. Increases in the rate of mitochondrial respiration in BAT and liver have been shown to correlate with increased energy expenditure for metabolic thermogenesis [25, 72–74]. Furthermore, BAT UCP_1_ expression was significantly higher in refed striped hamsters in the ICE-12 h/d and PCE groups than the CON group. Several studies have shown that the increase in thermogenesis in cold-exposed animals is based on a considerable up-regulated expression of UCP_1_ in BAT, playing an essential role in the regulation of body temperature [38, 40]. Up-regulation of BAT UCP_1_ expression has also been linked to significant decreases in body fat in several small mammals, which is thought to be effective at reducing adiposity [40, 68, 69, 75–77]. Treatments from various scientific fields have been shown to induce up-regulation of BAT UCP_1_ expression and decrease body fat content [27, 44, 78, 79]. Our findings suggest that short intermittent cold exposure (i.e. 2 h per day) may have little effect on mitochondrial respiration, UCP_1_ expression, and fat content of animals during the refeeding period. A more frequent or longer duration may be the most effective factor inducing up-regulation of BAT UCP_1_ expression, preventing fat accumulation.

The presence of thyroid hormone is thought to mediate BAT UCP_1_-based heat production because T_3_ potentiates UCP_1_ gene transcription [48–51]. We found that serum T_3_ level in striped hamsters was significantly higher in the ICE-12 h/d and PCE groups than the CON group. In mammals, thyroid hormone has been shown to improve thermoregulatory ability and increase metabolic thermogenesis [80–83]. For example, animals exposed or acclimated to cold temperatures usually have considerable increases in serum thyroid hormone levels and UCP_1_-based heat production in BAT. Our findings suggest that increased serum T_3_ levels may be necessary for UCP_1_-based heat production in BAT to cope with frequent or persistent cold exposure. Body fat accumulation was significantly reduced in the animals that were refed *ad libitum* due to thyroid hormone and UCP_1_-based heat production.

## Conclusions

When refed a high-fat diet following food restriction, striped hamsters recovered their lost weight. However, hamsters that were frequently and continuously exposed to cold temperature (ICE-12 h/d and PCE groups) had significantly less fat accumulation compared to those refed at 23 °C (CON group). This suggests that frequent exposure to cold effectively prevented fat accumulation during the period of body weight regain. Serum T_3_ levels, BAT UCP_1_ expression, and the rate of O_2_ consumption in BAT and liver were all significantly higher in the ICE-12 h/d and 24 h PCE groups, suggesting that TH mediated BAT UCP_1_-based heat production and liver heat production were possibly involved in the prevention of fat accumulation.

## Materials and methods

### Animals

Striped hamsters used in this study were offspring of a colony maintained at Wenzhou University, and supported with animals trapped in farmland in the center of Hebei province (115u139E, 38u129S) on the North China Plain. A randomized outbreeding protocol is used to maintain genetic diversity. Animals were housed individually in plastic cages (29 cm × 18 cm × 16 cm) with sawdust bedding and kept at a constant temperature of 23 ± 1 °C under a 12 h:12 h (light:dark, lights on at 08:00 h) photoperiod. Food (standard rodent chow; produced by Beijing KeAo Feed Co., Beijing, China) and water were provided *ad libitum*. All animal procedures performed in this research were in accordance with the ethical standards of the Wenzhou University Animal Care and Use Committee.

### Experimental design

**Fig. 1:**
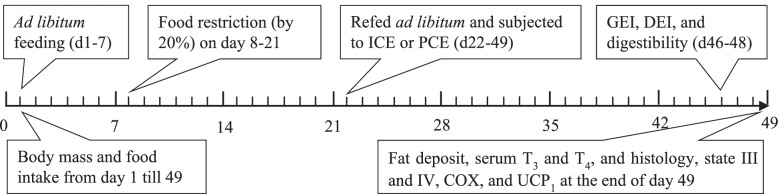
The feeding and cold exposure schedule. ICE = Intermittent cold exposure; PCE = persistent cold exposure; GEI = gross energy intake; DEI = digestive energy intake; T_3_ and T_4_ = 3,3′,5-triiodothyronine and 3,3′,5,5′ tetraiodothyroxyne, respectively; COX = cytochrome c oxidase; UCP_1_ = uncoupling protein 1

Fifty-six male hamsters (3–3.5 months of age) were randomly assigned to one of four groups: a control group at room temperature (CON), two intermittent cold exposure groups (ICE-2 h/d and ICE-12 h/d), and a persistent cold exposure group (PCE), with *n* = 14 for each group. All four groups of hamsters were kept at 23 °C for one week, during which time food and water was provided *ad libitum*. Food intake and body mass were measured daily for one week (baseline period, day 1 to 7) using a Sartorius balance (±0.1 g). Food intake was calculated by subtracting food residues mixed in the bedding materials from the difference between the initial food provided and the uneaten food the following day [16]. The average food intake throughout the week was then used to determine *ad libitum* food intake. After one week food was reduced by 20% for two weeks for all four groups of hamsters (food restriction period, day 8 to 21). A high-fat diet (fat 60%, carbohydrate 20% and protein 20%, total calories 22.0 kJ/g, Research Diet, D12492, USA) was then introduced and made available *ad libitum* for four weeks (refeeding period, day 22 to 49), throughout which the animals in the CON group were maintained at 23 °C, the ICE-2 h/d and ICE-12 h/d groups were exposed to 5 °C for 2 h and 12 h per day, respectively, and the animals in PCE group were maintained at 5 °C. A detailed feeding and cold exposure schedule is presented in Fig. 1.

**Fig. 2:**
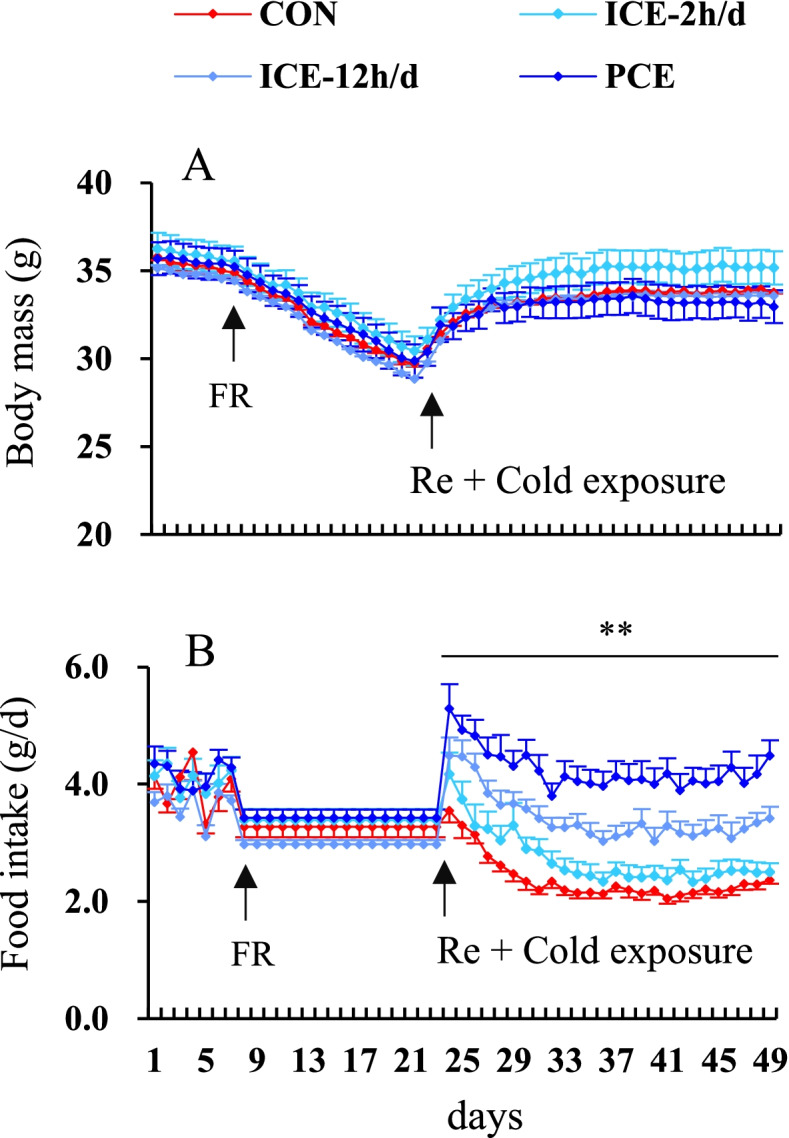
Body mass (**A**), and food intake (**B**) of striped hamsters that were subjected to food restriction and were refed *ad libitum*. Food intake was restricted by 20% of *ad libitum* for two weeks (FR, day 8–21), the animals were then refed *ad libitum* for four weeks (Re, day 22–49). Control animals (CON) were kept constantly at 23 °C throughout the experiment. During the *ad libitum* refeeding period, animals were intermittently exposed to 5 °C for 2 h per day (ICE-2 h/d), 12 h per day (ICE-12 h/d), or persistently exposed to 5 °C (PCE). Data are means ± SEM. ** = significant difference across the four groups (*P* < 0.01)

**Fig. 3:**
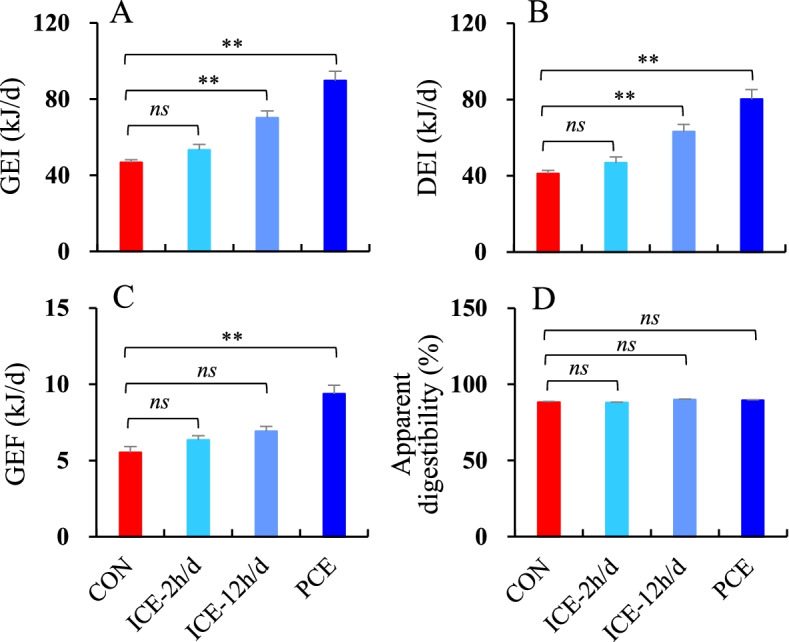
Gross energy intake, GEI (**A**); digestive energy intake, DEI (**B**); gross energy of feces, GEF (**C**); and apparent digestibility (**D**) of striped hamsters that were subjected to food restriction and were refed *ad libitum*. During *ad libitum* refeeding period, animals were maintained at 23 °C (CON), intermittently exposed to 5 °C for 2 h per day (ICE-2 h/d), 12 h per day (ICE-12 h/d), or persistently exposed to 5 °C (PCE). Data are means ± SEM. *ns* = non-significant difference between the two groups; ** = significant difference between the two groups (*P* < 0.01)

**Fig. 4:**
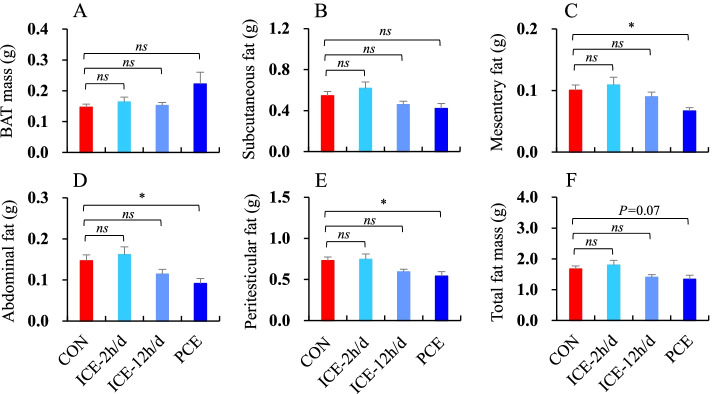
The mass of **A** brown adipose tissue, BAT; **B** Subcutaneous fat; **C** Mesentery fat; **D** Abdominal fat; **E** Peritesticular fat; and **F** Total fat of striped hamsters that were subjected to food restriction then refed *ad libitum*. During *ad libitum* refeeding period, animals were maintained at 23 °C (CON), intermittently exposed to 5 °C for 2 h per day (ICE-2 h/d), 12 h per day (ICE-12 h/d), or persistently exposed to 5 °C (PCE). *ns* = non-significant difference between the two groups; * = significant difference between the two groups (*P* < 0.05)

**Fig. 5:**
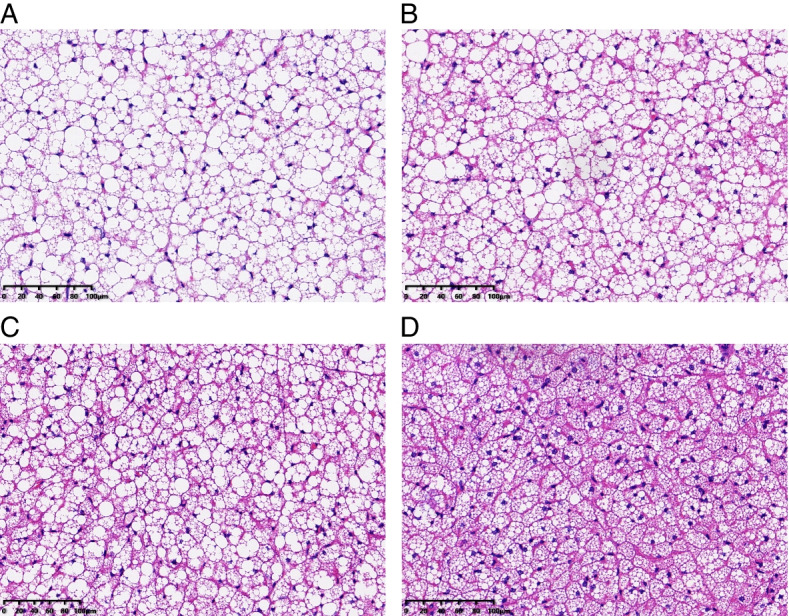
BAT specimens (H&E staining) of striped hamsters that were subjected to food restriction and then refed *ad libitum*. During *ad libitum* refeeding period, animals were maintained at 23 °C (**A**: CON), intermittently exposed to 5 °C for 2 h per day (**B**: ICE-2 h/d), 12 h per day (**C**: ICE-12 h/d), or persistently exposed to 5 °C (**D**: PCE)

**Fig. 6:**
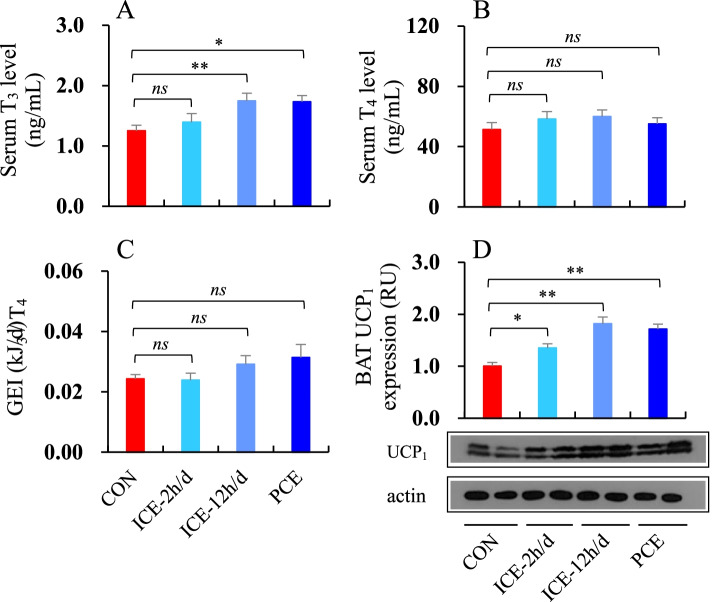
Levels of **A** serum T_3_; **B** T_4_; **C** the ratio of T_3_ to T_4_; and **D** BAT UCP_1_ expression of striped hamsters subjected to food restriction and refed *ad libitum*. During *ad libitum* refeeding period, animals were maintained at 23 °C (CON), intermittently exposed to 5 °C for 2 h per day (ICE-2 h/d), 12 h per day (ICE-12 h/d), or persistently exposed to 5 °C (PCE). *ns* = non-significant difference between the two groups; * = significant difference between the two groups (*P* < 0.05), ** = *P* < 0.01. The blots in panel D were cut prior to hybridisation with antibodies

**Fig. 7:**
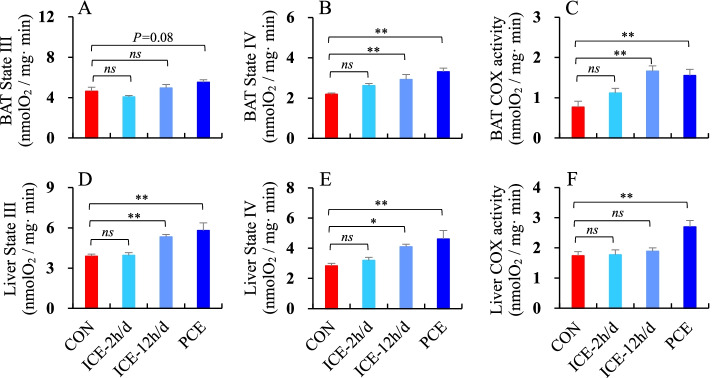
The rate of mitochondrial state III and state IV respiration and the activity of cytochrome c oxidase (COX) in BAT (**A**, **B** and **C**) and liver (**D**, **E** and **F**) in striped hamsters subjected to food restriction then refed *ad libitum*. During *ad libitum* refeeding period, animals were maintained at 23 °C (CON), intermittently exposed to 5 °C for 2 h per day (ICE-2 h/d), 12 h per day (ICE-12 h/d), or persistently exposed to 5 °C (PCE). *ns* = non-significant difference between the two groups; * = significant difference between the two groups (*P* < 0.05), ** = *P* < 0.01

### Energy intake and apparent digestibility

Gross energy intake (GEI) and apparent digestibility were measured in the last week of the high-fat refeeding period. As previously described by Wen et al. [84], a known quantity of food was provided and any uneaten food and orts in bedding material were collected 48 h later. Food and feces were separated manually after drying at 60 °C to constant mass. The gross energy content of feces was determined using an IKA C2000 oxygen bomb calorimeter (IKA, Germany). GEI, digestive energy intake (DEI), gross energy of feces (GEF) and apparent digestibility were calculated as follows [85, 86].

$${\displaystyle \begin{array}{c}\mathrm{GEI}\ \left(\mathrm{kJ}/\mathrm{d}\right)=\mathrm{food}\ \mathrm{intake}\ \left(\mathrm{g}/\mathrm{d}\right)\times \mathrm{dry}\ \mathrm{matter}\ \mathrm{content}\ \mathrm{of}\ \mathrm{food}\ \left(\%\right)\times \mathrm{gross}\ \mathrm{energy}\ \mathrm{content}\ \mathrm{of}\ \mathrm{food}\ \left(\mathrm{kJ}/\mathrm{g}\right)\\ {}\mathrm{GEF}\ \left(\mathrm{kJ}/\mathrm{d}\right)=\mathrm{dry}\ \mathrm{feces}\ \mathrm{mass}\ \left(\mathrm{g}/\mathrm{d}\right)\times \mathrm{gross}\ \mathrm{energy}\ \mathrm{content}\ \mathrm{of}\ \mathrm{feces}\ \left(\mathrm{kJ}/\mathrm{g}\right)\\ {}\begin{array}{c}\mathrm{DEI}\ \left(\mathrm{kJ}/\mathrm{d}\right)=\mathrm{GEI}-\mathrm{GEF}\\ {}\mathrm{Apparent}\ \mathrm{digestibility}\ \left(\%\right)=\mathrm{DEI}/\mathrm{GEI}\times 100\%\end{array}\end{array}}$$

### Body fat deposit

Following the high-fat refeeding period, animals were euthanized by decapitation. Trunk blood was collected for hormonal measurements. Serum was separated from each blood sample and stored at −20 °C. Scapular BAT and liver were removed and immediately frozen in liquid nitrogen and stored at −80 °C until analysis. Subcutaneous fat, perirenal fat, mesenteric fat, and abdominal fat (but not periovarian fat), were collected and weighed (to ±1 mg). Total body fat deposit was calculated from the summed fat deposit described above.

### BAT specimens stained with H&E

After removal, scapular BAT (about 3 x 3 x 3 mm^3^) of sixteen animals (*n* = 4 per group) was immediately washed with PBS solution twice and stored in paraformaldehyde. 24 h later, the BAT was washed, dehydrated in graded alcohol concentrations, cleared in xylol and embedded in paraffin. The paraffin blocks were cut serially to provide 5 μm-thick sections. Serial sectioning was performed until reaching the maximum number of sections possible. Sections representative of each specimen were selected and stained with hematoxylin-eosin dyestuff. The stained slice was dehydrated with pure alcohol and made transparent using xylene. The transparent slice was then placed onto a slide and sealed with a coverslip. Images were captured using a microscope (ECLIPSE E100, NIKON, Japan) coupled to a video camera (K-Viewer 1.5.5.10 x64, KFBIO, China). Ten slices were randomly selected from each animal for the histometric analysis, and one image representative of its respective group was selected to present in this paper.

### Serum levels of triiodothyronine (T_3_) and thyroxine (T_4_)

Serum T_4_ and T_3_ concentrations were determined using I^125^ RIA kits (Beijing North Institute of Biological Technology, Beijing, China), which has been previously validated for striped hamsters [87]. The intra- and inter-assay coefficients of variation were 2.4 and 8.8% for T_3_, respectively, and 4.3 and 7.6% for T_4_, respectively [29].

### Mitochondrial state IV, state III respiration and cytochrome C oxidase (COX) activity

Mitochondrial state IV, state III respiration and COX activity in BAT and liver were evaluated with the rate of oxygen consumption of the tissue homogenates. We used 0.1 g/mL mitochondria isolation liquid (250 mM sucrose, 5 mM Tris, 1 mM MgCl_2_ ·6H_2_O, 0.5 mM EDTA and 0.5 mg/mL bovine serum albumin, pH 7.4) at 4 °C to prepare tissue homogenates [55]. Oxygen consumption was measured polarographically with a Clark-type electrode (Hansatech Instruments, DW-1, England) at 30 °C in a medium containing 225 mM sucrose, 50 mM Tris, 5.0 mM MgCl_2_·6H_2_O, 1.0 mM EDTA and 5.0 mM KH_2_PO_4_, pH 7.4. Substrates used were 5.0 mmol/L succinate and 3.75 mol/L rotenone. Measurements were performed in the absence (state IV) and presence (state III) of 1.0 mmol/L ADP [88]. The COX activities were measured at 30 °C in 2.0 ml of respiration medium (7.5 mM KH_2_PO_4_, 3.75 mM ascorbic acid and 0.3 mM TMPD (N, N, N′, N′-Tetramethyl-p-phenylenediamine dihydrochloride), pH 7.4), using a Clark electrode, as described previously [89].

### Western blot analysis of UCP_1_

BAT uncoupling protein 1 (UCP_1_) concentrations were measured as described previously [77]. BAT was lysed in RIPA buffer (0.5% NP-40, 0.1% sodium deoxycholate, 150 mM NaCl, 50 mM Tris-HCl, pH 7.5) supplemented with phosphatase inhibitor cocktails. Protein extracts were diluted in 5× sample buffer (50 mM Tris at pH 6.8, 2% SDS, 10% glycerol, 5% β-mercaptoethanol, and 0.1% bromophenol blue), and were separated in a discontinuous SDS-polyacrylamide gel (12% running gel and 5% stacking gel) and subsequently transferred onto a PVDF membrane (millipore, IPVH00010). The blotting membranes were blocked with 5% (wt/vol) milk powder and incubated overnight at 4 °C with the primary antibody β-actin (Servicebio GB12001; 1:3000) and UCP_1_ (proteintech 23,673–1-AP, 1:1000). Secondary antibody (anti-rabbit IgG HRP conjugate; 1:3000; Servicebio GB23303) was added and Super Signal Western Blot Enhancer (Thermo Scientific) was used to visualize protein bands. Blots were analyzed with Bio-Rad Quantity One and normalized to β-actin.

### Statistical analysis

Data are expressed as means ± SE and analyzed with SPSS 21.0 statistical software. All variables were tested for normality with the Kolmogorov-Smirnov test, which confirmed that all data was normally distributed. Body mass, food intake, GEI, DEI, serum T_3_ and T_4_ levels, mitochondrial state IV, state III respiration, and COX activity in BAT and liver, as well as UCP_1_ protein expression in BAT, were analyzed using one-way ANOVA. The statistical significance of specific between-group differences was assessed using Tukey’s post hoc tests where required. Correlation coefficients between different variables were estimated using Pearson’s correlation coefficient. Significance (two-tails) was set at *P < .05*.

## Supplementary Information

**Additional file 1.**

**Additional file 2.**

## Data Availability

Datasets are available from the corresponding author upon reasonable request.

## Abbreviations

UCP_1_: Uncoupling protein 1
BAT: Brown adipose tissue
T_3_: 3,3′,5-triiodothyronine
T_4_: 3,3′,5,5′ tetraiodothyroxyne
GEI: Gross energy intake
DEI: Digestive energy intake
GE: Gross energy of feces
COX: Cytochrome C oxidase
