# Supplementary figures and images for: Cold exposure prevents fat accumulation in striped hamsters refed a high-fat diet following food restriction
Source: BMC Zool. 2022 Apr 18;7:19. doi: 10.1186/s40850-022-00122-z (PMC10127302; doi:10.1186/s40850-022-00122-z)

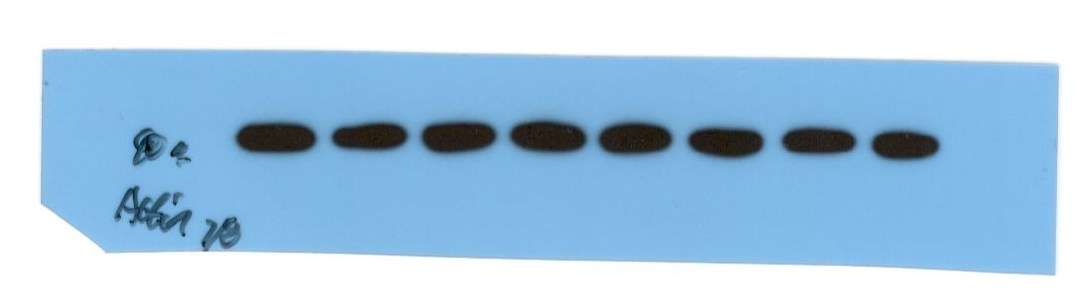

Supplement: Supplementary file 1 — Additional file 1. [file 40850_2022_122_MOESM1_ESM.tif]

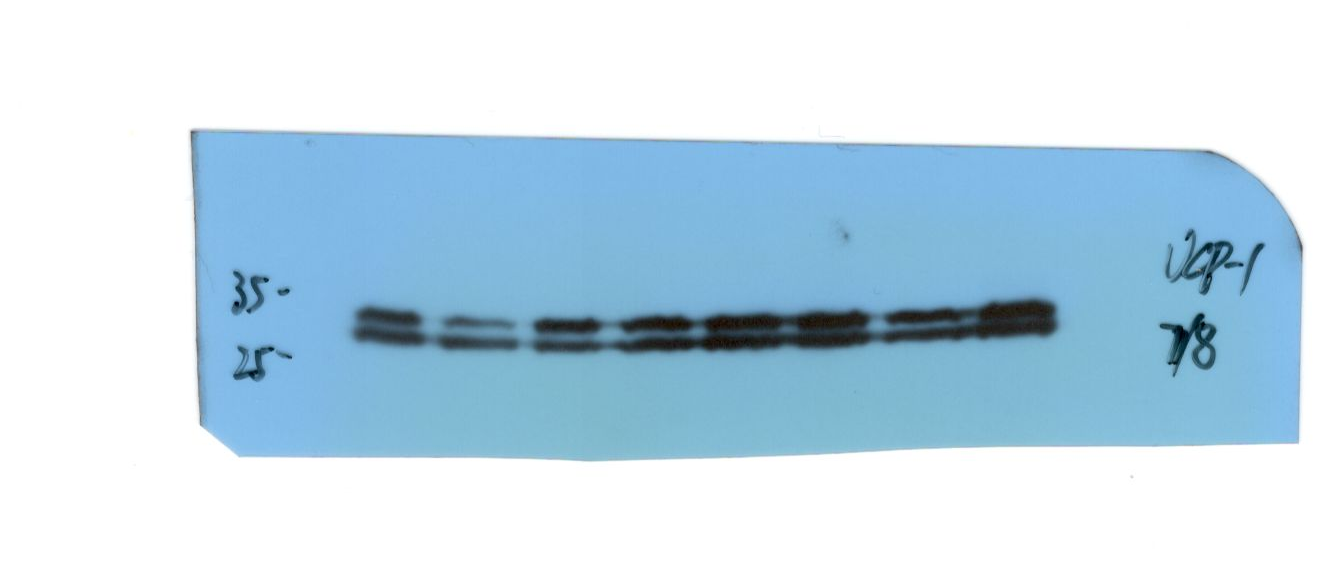

Supplement: Supplementary file 2 — Additional file 2. [file 40850_2022_122_MOESM2_ESM.tif]
